# The Role of Bleeding History and Clinical Markers for the Correct Diagnosis of VWD

**DOI:** 10.4084/MJHID.2013.051

**Published:** 2013-07-12

**Authors:** Alberto Tosetto

**Affiliations:** Hemophilia and Thrombosis Center, Hematology Department, S. Bortolo Hospital, Vicenza, Italy

## Abstract

Quantification of the bleeding severity by use of bleeding assessment tools (BAT) and bleeding score (BS) has been consistently shown to improve the clinical diagnosis of von Willebrand disease (VWD) while helping researchers establish phenotype/genotype correlations. Subjects with a BS equal or higher than 3 may be consider having a bleeding tendency, and should be referred for a laboratory investigation, particularly for VWD. In the diagnosis of type 1 VWD, the use of the BS has been shown to be highly specific (>95%) with reported sensitivities ranging from 40 to 100%. The BS is related to all available measurements of von Willebrand factor activity, including the PFA-100 closure time. Therefore, in clinical practice the use of BAT should always be the first step to standardize the assessment of patients with suspected VWD. The use of the recent ISTH consensus BAT is suggested to harmonize the collection of bleeding symptoms in patients with a suspected or confirmed hemostatic disorder, particularly VWD. The ISTH BAT is also coupled with a Web-based repository of bleeding symptoms, therefore providing an integrated framework for collaboration in the field of clinical evaluation of VWD and mild bleeding disorders.

## Introduction

The diagnosis of von Willebrand disease (VWD) may be consider as the epitome of the difficulties encountered in the diagnosis of mild bleeding disorders. In contrast with patients having a severe bleeding disorder, who usually have a clear-cut clinical and laboratory phenotype, VWD patients usually report a mild bleeding history and have a partial reduction of plasma von Willebrand factor levels. This clinical and laboratory pattern is consistent with the heterozygous transmission of the defect, with circulating von Willebrand factor (VWF) around 30–40 IU/dL without evident structural abnormalities in the most common variety of VWD, type 1 VWD.^1^

In the last years, some diagnostic criteria have been proposed to tentatively standardize VWD diagnosis.^2^ A “definite” diagnosis of type 1 VWD requires the presence of significant mucocutaneous bleeding (this latter being presence of at least two symptoms or of one requiring blood transfusion), reduced VWF levels (>2SD below the mean) and at least another family member with reduced VWF. The diagnosis of “possible” type 1 VWD requires the presence of reduced VWF levels and either significant mucocutaneous bleeding or at least another family member with reduced VWF levels.

The “definite” and “possible” diagnostic categories criteria were clearly modeled after the definition of VWD as an autosomal dominant bleeding disorder due to the reduction of circulating VWF. Although these criteria may provide a common diagnostic background, they also have several weaknesses. First, the definition for “a significant bleeding history” is uncertain, since the high frequency of bleeding symptoms reported in healthy controls challenges the idea that a bleeding disorder should be sought in every subject referring some. For instance, profuse menstrual bleeding has been reported to occur at least once during the fertile period in up to 44% of women; epistaxis in 5–36% of children; and at least one hemorrhagic symptom is reported by 40–50% of men and 50–60% of women.^3^–^7^ Such figures were recently confirmed in a very large investigation (n=500) on the prevalence of bleeding symptoms, in which menorrhagia was reported by 47% of women, epistaxis by 25%, easy bruising by 18%, and prolonged bleeding after tooth extraction by 18% of the whole sample^8^

Second, incomplete penetrance and variable expressivity due to environmental (e.g., age, stress, pregnancy, menstruation, drugs) or epigenetic (e.g., ABO blood group) factors may significantly interfere with the laboratory diagnosis of type 1 VWD patients.^9^ Finally, these provisional have never been validated and their sensitivity and specificity in a clinical setting remain unknown.

In this brief review, we will therefore focus on the role of bleeding history in the diagnosis of VWD, as apparent from recent studies.

## Bleeding history and laboratory data for the diagnosis of VWD

The process of VWD diagnosis (or, in a wider context, the evaluation of a possible mild bleeding disorder) may start either from the clinical assessment of a subject referred for bleeding symptoms (i.e., a *symptom-driven* diagnosis) or from the laboratory study of an abnormal laboratory test (i.e., a *laboratory-driven* diagnosis, such as in a preoperative screening).^10^ The laboratory-driven diagnosis is often unsatisfactory, because of the poor correlation between plasma VWF levels and severity of bleeding symptoms, resulting in a scarce predictive value of VWF measurement. In fact, even assuming in the general population a VWD prevalence as high as 1%,^11^ plasma VWF levels below 20 IU/dL would be required to diagnose VWD based on laboratory data alone.^12^,^13^ Therefore, laboratory measurement of VWF levels in unselected patients is not effective, further confirming the notion that preoperative screening tests are of minimal value in the prediction of post-surgical bleeding.^14^

The proper evaluation of the bleeding symptoms referred by the patient is therefore the cornerstone of a meaningful, *symptom-driven* diagnosis. Several investigators have described the use of bleeding questionnaires, mainly for the purpose of predicting bleeding before surgery,^15^,^16^ but only recently there have been some efforts to quantitatively describe the severity of a mild bleeding disorder and use bleeding severity as a tool for diagnosis.^17^,^18^ The quantitative assessment of bleeding symptoms is a two-sided process, first requiring collection of the bleeding history and then the interpretation of data using pre-established criteria.

To this purpose, a standardized bleeding questionnaire is usually administered by trained personnel: a physician (or a nurse) interviews the patient about both presence and absence of bleeding symptoms (i.e., all instances of surgery or tooth extraction should also be recorded, even if no hemorrhages occurred), and has been already extensively been reviewed.^19^,^20^ Second, collected data must be interpreted to verify if the bleeding history is compatible with a bleeding disease, using a predefined interpretation grid. Collectively, a standardized bleeding questionnaire and its interpretation grid are defined as a “Bleeding Assessment Tool” (BAT). In most cases, a BAT could be used to generate a “bleeding score” (BS), which is generated by summing the severity of all bleeding symptoms reported by a subject, as graded according to the interpretation grid. Several BATs have been proposed, sometimes with minimal variations or adaptations, for instance to account for pediatric-specific symptoms (see 21 for a recent review). To improve standardization, the International Society of Thrombosis and Haemostasis (ISTH) has recently endorsed the development of a consensus BAT to collect and interpret clinically relevant bleeding symptoms (see the ISTH web page at http://www.isth.org/members/group.asp?id=100549), and it is strongly hoped that this BAT will harmonize results from different studies in the next years.^22^

## The use of bleeding assessment tools for the description and diagnosis of VWD

The first use of a BAT in the description of the VWD bleeding phenotype was in the seminal International Multicenter Study (IMS), in which bleeding symptoms were collected in 42 obligatory carriers of type 1 VWD and compared with 215 control subjects; VWD obligatory carriers were studied to avoid any possible bias resulting from selection by symptom.^18^ In the study, each bleeding symptom received a score ranging from 0 (for absence) to 3. Grade 1 was given when a patient reported presence of bleeding, grade 2 if the symptom required evaluation by a physician but no active intervention, grade 3 if there was some kind of intervention by the physician. In normal controls, the BS was <3 in males and <5 in females; using this cutoff, the BS showed a diagnostic sensitivity (64.3%) higher than a criterion based on number of bleeding symptoms.

In a subsequent refinement, the number of possible grades for each bleeding symptom was increased, ranging from −1 to 4, basically to improve the sensitivity of the bleeding score for possibly influencing biological variables in the setting of the European MCMDM VWD-1 Study.^23^,^24^ In this revised BAT, an additional grade (4) was added to account for the most dramatic presentations (requiring blood transfusion or surgery to control bleeding) and a −1 grade was introduced to highlight the importance of the absence of bleeding despite a hemostatic challenge, such as after surgery or tooth extraction. A comparison between the two BATs (the one used in the IMS and the “−1” used in the MCMDM VWD-1 Study) did not show appreciable differences in the diagnostic efficiency, however,^25^ and for this reason the use of the “−1” has been dropped in the consensus ISTH BAT.^22^

The MCMDM VWD-1 Study was the first study in which the BS in VWD patients was shown to correlate with the severity of the hemostatic defect, both as VWF:Ag, VWF:Rco and FVIII:C; furthermore, different levels of bleeding severity could be demonstrated in obligatory carriers, affected family members and normal controls with increased BS in older patients: all these findings have been confirmed by the recent WiN study on VWD in the Netherlands.^26^ The BS did correlate also with the PFA-100 closure time, a measure of VWF/platelet interaction, in VWD patients.^27^ The correlation between a symptom-derived bleeding score and the severity of the hemostatic defect has been also demonstrated in a cohort of 114 women with various bleeding disorders, including VWD.^28^ The use of the BS as a sensitive tool for the study of genotype/phenotype correlations has been recently exploited by Flood et al, who used the bleeding score to demonstrate that the D1472H sequence variation of the VWF gene does not result in any increased bleeding risk.^29^

The clinical utility of a pediatric-specific BAT for the diagnosis of VWD has been demonstrated in a small series, with sensitivity and specificity of 83 and 79%, respectively.^30^ In a larger series of 100 VWD affected children, the BS ranged from 0 to 29 (median value, 7.0) whereas it ranged from 1–2 in normal controls (median 0).^31^

Finally, The BS has been prospectively tested for the diagnosis of patients referred for evaluation of bleeding symptoms and/or abnormal laboratory screening tests in a secondary setting.^32^ A clinical prediction guide based on BAT and aPTT could be useful to exclude patients with suspected MBD in a low-prevalence (screening) setting. Assuming a prevalence for MBD in the general population around 1%, a normal BS (<=3) had a very high negative predictive value (99.2%). The positive predictive value in patients referred for hemostatic or familial evaluation at second level clinics was estimated to be 71.0 and 77.5% (assuming a MDB prevalence of 20% and 50%, respectively).

## An integrated, Bayesian approach to VWD diagnosis

One of the benefits of using the BS could be the possibility of establishing likelihood ratios of VWD for each level of BS, and to integrate the information coming from the bleeding history with those obtained from VWF measurement or family history.^33^ Such data have been provided by the European MCMDM-1VWD study, and demonstrate that the likelihood of VWD increase approximately in an exponential way with each unit increase of BS,^13^ resulting in a very unlikely VWD diagnosis for a BS below 0 but a very likely VWD diagnosis for a BS above 4. Our group tried to integrate information coming from clinical, laboratory and family data into a single value (the final probability of having VWD^12^). With this approach, the physician collects data about the bleeding history in the patient (summarized in a BS) and measures VWF level in the patients and in as much first relatives as possible (to detect relatives with VWF levels below the normal range). This information is translated into likelihood ratios for bleeding history, VWF level and family data, respectively, and then used to compute the final probability of having VWD. For instance, the presence of VWF levels below 40 IU/dL in at least two family members (including the proband) and of a BS at least of 1 in the proband resulted in a final odd of VWD of 2.0 (or a final probability of VWD of 66%). Therefore, rather than using fixed criteria with unknown sensitivity or specificity, physicians may compute the probability of VWD for each given subjects of a particular family. This approach appears to be more flexible, but it still requires further validation before being proposed for a widespread use.

## When a patient should be considered for evaluation of VWD?

Although the best approach of the individual case remains a choice of the physician, depending on both the clinical and laboratory findings, some scenarios could be broadly considered.

### The patient with bleeding symptoms

A bleeding score above 3 could be generally considered as suggestive of a bleeding diathesis,^10^ but slightly lower (e.g., 2–3 in males and 3–4 in females) bleeding scores may be still be for a laboratory investigation of VWD, at least in very young patients (see below). It is of paramount importance to carefully distinguish between trivial bleeding symptoms, which are frequently reported by normal subjects, and clinically relevant bleeding symptoms that should be more carefully considered. Criteria for clinically relevant bleeding symptoms have been reported by the ISTH consensus paper and are reported in Table 1.

### The pediatric patient

The pediatric patient with bleeding symptoms should be carefully evaluated because he/she is usually referred for only scarce manifestations that would be otherwise dismissed as “trivial” in an adult. Data from other family members should always be collected, since bleeding in other relatives may be frequently reported for autosomal dominant disorders.^17^ The family history may be however negative for recessive disorders, and a complete laboratory evaluation of the pediatric patient should be performed in doubtful cases. Among the latter, there is the patient (frequently a young child) with the serendipitous finding of a prolonged aPTT; we usually always test for reduced VWF:Ag in such cases.

### The asymptomatic patient with a family history of bleeding

Counseling an asymptomatic relative of a patient with known VWD (the proband) could be particularly difficult. Even if the same type of deficiency observed in the proband is identified in a relative, there are no clues that may be used to predict bleeding in such patients. Selection bias, presence of circumstantial factors (e.g., aspirin use), co-inheritance of other mild bleeding disorders may significantly worsen the bleeding diathesis in the proband, and extreme caution should be used before labelling an asymptomatic patient as “VWD affected”. Therefore, even before evaluating for a specific laboratory defect, reassurance of the asymptomatic patient is always advisable.

## Conclusions

In the last decade, the use of standardized tools for the assessment of bleeding has provided a significant impact for both the diagnosis and study of von Willebrand disease. The recently proposed ISTH BAT has been developed to ensure compatibility with previous investigations, and aims at standardizing the various interpretation grids (and the resulting bleeding scores) while collecting more detailed clinical data on the bleeding patient.^22^ The ISTH BAT is also coupled with an electronic repository, set up and kept by the Rockefeller University Center for Clinical and Translational Science, designed to collect and possibly merge information on bleeding symptoms and rates in different patient populations. As clinical data on VWD accumulates, it is hoped that more insights on the genetic and environmental risk factors modulating bleeding risk in VWD will be gained in the next future, allowing better knowledge and treatment of an intricate bleeding disorder.

## Figures and Tables

**Table 1:** Minimal criteria defining a non-trivial bleeding symptom (after Rodeghiero et al^22^)

Symptom	Criteria
**Epistaxis:**	Any nosebleed that causes interference or distress with daily or social activities.
**Cutaneous bleeding**	Bruises are considered significant when 5 or more (> 1cm) in exposed areas.
**Minor cutaneous wound:**	Any bleeding episode caused by superficial cuts (e.g., by shaving razor, knife, or scissors) or that requires frequent bandage changes.
**Oral cavity bleeding:**	Gum bleeding should be considered significant when it causes frankly bloody sputum and lasts for 10 minutes or longer on more than one occasion. Tooth eruption or spontaneous tooth loss bleeding should be considered significant when it requires assistance or supervision by a physician, or lasts at least 10 minutes. Bleeding occurring after bites to lips, cheek, and tongue should be considered significant when it lasts at least 10 minutes or causes a swollen tongue or mouth.
**Tooth extraction:**	Any bleeding occurring after leaving the dentist’s office and requiring a new, unscheduled visit or prolonged bleeding at the dentist’s office causing a delay in the procedure or discharge.
**Surgical bleeding:**	Any bleeding judged by the surgeon to be abnormally prolonged, that causes a delay in discharge, or requires some supportive treatment.
**Menorrhagia:**	Any bleeding that interferes with daily activities such as work, housework, exercise or social activities during most menstrual periods.
